# Spontaneous sigmoid perforation and subsequent ruptured hepatic aneurysms in suspected vascular type Ehlers–Danlos syndrome: a case report and comprehensive literature review

**DOI:** 10.1093/jscr/rjae726

**Published:** 2024-11-25

**Authors:** SaeRam Oh, Aaron D Hudnall, Caitlin A Fitzgerald

**Affiliations:** Department of Surgery, Brody School of Medicine at East Carolina University, Greenville, NC 27834, United States; Department of Surgery, Division of Trauma and Acute Care Surgery, Brody School of Medicine at East Carolina University, Greenville, NC 27834, United States; Department of Surgery, Division of Trauma and Acute Care Surgery, Brody School of Medicine at East Carolina University, Greenville, NC 27834, United States

**Keywords:** vascular type Ehlers–Danlos syndrome, spontaneous bowel perforation, vascular aneurysm rupture

## Abstract

Ehlers–Danlos syndrome (EDS) is an inherited disorder of collagen creation and function which can affect many organs. Surgical management of EDS spectrum remains a significant challenge for surgeons, including the vascular type of EDS (vEDS). There do not exist specific guidelines for the management of vEDS, which proves difficulty given the devastating pathology and potential outcomes. This case report emphasizes the need for further research in many areas including the need for certain screenings to identify any vascular aneurysms or dissections prior to rupture, as well as asking should there be a screen for this gene mutation in COL3A1 included at birth. Our case report is one of few reports that link the spontaneous colonic rupture that may trigger the subsequent vascular catastrophe leading to devastating mortality.

## Introduction

Ehlers–Danlos syndrome (EDS) is an inherited disorder of collagen creation and function which can affect many organs. The complications of EDS include joint problems, chronic fatigue and pain, cardiovascular anomalies, and even complications as serious as visceral and arterial rupture. Although there are 13 different variants with six types, EDS is typically a clinical diagnosis with genetic testing allowing for identification of the specific variant [1].

The vascular form of EDS (vEDS) is an autosomal dominant mutation in the COL3A1 gene affecting the pro-alpha 1 chain of type III procollagen [2, 3]. There are major and minor clinical criteria including arterial or digestive rupture, thin and translucent skin, easy bruising, characteristic facial appearance, tendon rupture, spontaneous pneumothorax, and positive family history [4]. It is estimated that the vEDS represents 5–10% of EDS spectrum [5, 6]. Median age of death for vEDS is 50 and most common cause of mortality is due to arterial ruptures [4]. The overall care of vEDS patients focuses on treatment of complications rather than prevention and avoiding secondary complications after interventions [4]. Here, we present a case with suspected diagnosis of vEDS who presents with spontaneous bowel perforation with subsequent catastrophic vascular complication.

## Case report

**Figure 1:**
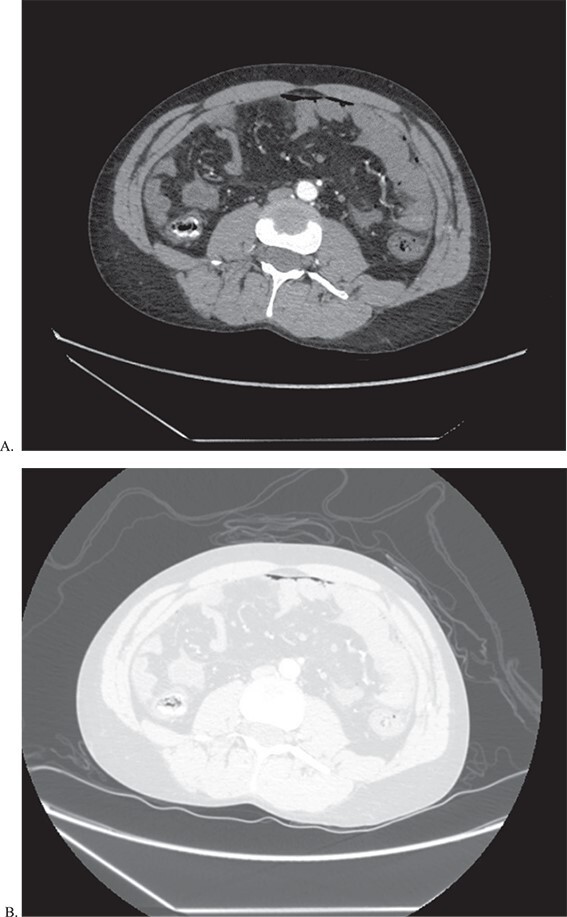
CT of abdomen and pelvis in both abdominal (A) and lung (B) window to show pneumoperitoenum as well a thickened loops of bowel.

A 21-year-old male presents to the emergency department (ED) with acute onset abdominal pain. The patient has a clinical history suggestive of EDS including aortic insufficiency, joint instability, spontaneous pneumothorax, acquired foot deformity, and iliofemoral deep vein thrombosis and has been undergoing work up for EDS. Initial CT of the abdomen and pelvis (Fig. 1) revealed pneumoperitoneum and free fluid suggestive of a perforated hollow viscus without any visceral aneurysms. Therefore, the patient was taken to the operating room (OR) emergently.

During the operation, a small perforation in the sigmoid colon was identified with gross feculent contamination. The patient underwent sigmoid colectomy with primary hand sewn anastomosis and diverting loop ileostomy. The patient recovered well post operatively and was discharged from the hospital on post-operative day (POD) 5. The patient was closely followed up in the outpatient setting on POD 7 and was recovering well.

**Figure 2:**
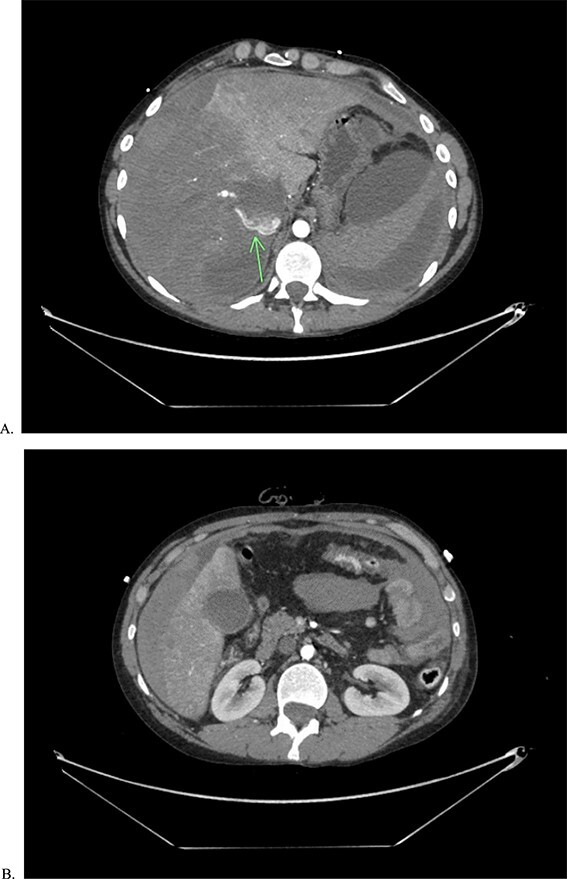
CTA of abdomen and pelvis showing the large contrast extravasation (A) as well as significant amount of free fluid in the abdomen (B).

He returned to the ED on POD 9 with episodes of emesis and abdominal pain. En route, he was hypotensive and had a coughing spell with witnessed evisceration. The initial workup included a FAST (focused assessment with sonography in trauma) exam which was positive for free fluid in the abdomen. Massive transfusion protocol (MTP) was initiated, and the patient was sent for CT angiogram of the chest, abdomen, and pelvis en route to the OR. The scan revealed a large area of contrast extravasation in the right hepatic lobe with a significant amount of free fluid (Fig. 2).

In the OR, his hemodynamic status did not improve despite hepatic packing and MTP, thus a resuscitative endovascular balloon (REBOA) in Zone I was placed. His hemodynamics improved immediately. A temporary abdominal closure was placed, and the patient was transported to IR where he underwent embolization of the right hepatic artery due to ruptured aneurysms. After embolization, the patient was transferred to the surgical intensive care unit and resuscitation continued. He eventually passed away on POD 13 from the initial operation, POD 4 from the second admission operation due to multi-organ failure as well as profound coagulopathy that was difficult to reverse despite multiple trips to the OR.

## Discussion

Spontaneous hollow viscous perforation is a well-known complication occurring in patients with the diagnosis of vEDS. The pathophysiology behind it is thought to be perforation secondary to poor tensile strength in the colonic wall due to defective type III collagen that is mostly found on skin, blood vessels, and internal organs [6, 7]. Mortality associated with the hollow viscous rupture is reported as high as 12 to 20% and it is one of the common presentations of vEDS. The most common location for perforation is the sigmoid colon, estimated to account for 80% of the cases [7]. Reperforations of the GI tract, anastomotic rupture or leaks, fistula formation, hernia formation and poor wound healing leading to dehiscence and evisceration are common post-operative complications [6].

Surgical precautions have been described including careful and minimal tissue handling, use of soft jawed or shodded vascular clamps, buttressing of suture lines, and leaving skin sutures in place longer than normal [8]. According to Menni *et al.*, after systemic reviews of case reports on spontaneous colonic perforations in vEDS, Hartmann’s procedure is a tested and relatively secure choice [6]. Interestingly, there are multiple systemic reviews for spontaneous ruptured hollow viscous organ as well as for ruptured arterial aneurysms; however, there is paucity in the literature for a systemic review of the relationship between spontaneous colon rupture with subsequent arterial pseudoaneurysm or dissection complications.

To this date, only one case report has been published that describes a vascular catastrophe following a spontaneous colonic perforation [9]. The theory behind this vascular complication after perforation or frank peritonitis is thought to be due to an increase in collagenase activity. One of the reviews by Speake *et al.* reported many causes of perioperative deaths after spontaneous colonic perforation, including spontaneous rupture of iliac arteries, renal arteries, retroperitoneal hemorrhage, and multi-organ failure.

Our case report, as well as the one in Kakinuma *et al.*, shows that common complications seen in vEDS may be related to pathophysiology that has yet to be fully elucidated. One complication may warrant closer monitoring throughout the patient’s lifetime. This opens area of further investigation for a prospective or a full comprehensive, multicentered retrospective reviews on patients with diagnosis or suspected vEDS who presents with spontaneous ruptured hollow viscous or arterial aneurysm that subsequently develop other complications. This case report emphasizes the need for further research in many areas including the need for certain screenings to identify any vascular aneurysms or dissections prior to rupture, as well as asking should there be a screen for this gene mutation in COL3A1 included at birth. Additional areas of further investigation include nutritional needs for those with defective collagen formation to help with the wound healing and integrity of tissues.

## References

[1] Shabani M , Abdollahi A, Brar BK, et al. Vascular aneurysms in Ehlers-Danlos syndrome subtypes: a systematic review. Clin Genet 2023;103:261–7. 10.1111/cge.14245. 36210598 PMC12172680

[2] Cortini F , Marinelli B, Seia M, et al. Next-generation sequencing and a novel COL3A1 mutation associated with vascular Ehlers-Danlos syndrome with severe intestinal involvement: a case report. J Med Case Rep 2016;10:303. 10.1186/s13256-016-1087-0. 27799058 PMC5088665

[3] Moramarco LP , Capodaglio CA, Quaretti P, et al. Multivessel endovascular therapy for undiagnosed vascular type Ehlers-Danlos syndrome. Successful percutaneous transcatheter coil embolization of hepatic artery pseudoaneurysm with stenting of right renal and iliac arteries in emergency setting. BJR Case Rep 2020;6:20200025. 10.1259/bjrcr.20200025. 33299587 PMC7709055

[4] Germain DP . Ehlers-Danlos syndrome type IV. Orphanet J Rare Dis 2007;2:32. 10.1186/1750-1172-2-32. 17640391 PMC1971255

[5] Charlier P , Germain DP, Jeunemaître X, et al. Sudden death associated to vascular Ehlers-Danlos syndrome. A case report. Leg Med (Tokyo) 2011;13:145–7. 10.1016/j.legalmed.2010.12.004. Erratum in: *Leg Med (Tokyo)*. 2013;15:288. Delisle, Stanislas Grassyn [corrected to Grassin-Delyle, Stanislas]. 21269862

[6] Menni A , Tzikos G, Sarafis A, et al. Bowel perforation in vascular Ehlers-Danlos syndrome: case report and comprehensive review. J Pers Med 2023;13:1247. 10.3390/jpm13081247. 37623497 PMC10455523

[7] Speake D , Dvorkin L, Vaizey CJ, et al. Management of colonic complications of type IV Ehlers-Danlos syndrome: a systematic review and evidence-based management strategy. Colorectal Dis 2020;22:129–35. 10.1111/codi.14749. 31260161

[8] Parfitt J , Chalmers RTA, Wolfe JHN. Visceral aneurysms in Ehlers-Danlos syndrome: case report and review of the literature. J Vasc Surg 2000. 31:1248–51. 10.1067/mva.2000.105667 10842163

[9] Kakinuma D , Yamada T, Kanazawa Y, et al. A case of vascular Ehlers-Danlos syndrome with a ruptured hepatic artery after surgical treatment of peritonitis caused by the perforation of the colon. Surg Case Rep 2021;7:74. 10.1186/s40792-021-01156-0. 33755833 PMC7988026

